# Activation of TRPV4 Regulates Respiration through Indirect Activation of Bronchopulmonary Sensory Neurons

**DOI:** 10.3389/fphys.2016.00065

**Published:** 2016-02-29

**Authors:** Qihai (David) Gu, Charles R. Moss, Kristen L. Kettelhut, Carolyn A. Gilbert, Hongzhen Hu

**Affiliations:** ^1^Division of Basic Medical Sciences, Mercer University School of MedicineMacon, GA, USA; ^2^Department of Anesthesiology, The Center for the Study of Itch, Washington University School of Medicine in St. LouisSt. Louis, MO, USA

**Keywords:** alveolar macrophage, bronchopulmonary sensory neuron, pulmonary chemoreflex, rapid shallow breathing, transient receptor potential vanilloid receptor 4

## Abstract

Transient receptor potential vanilloid receptor 4 (TRPV4) is a calcium-permeable non-selective cation channel implicated in numerous physiological and pathological functions. This study aimed to investigate the effect of TRPV4 activation on respiration and to explore the potential involvement of bronchopulmonary sensory neurons. Potent TRPV4 agonist GSK1016790A was injected into right atrium in anesthetized spontaneously breathing rats and the changes in breathing were measured. Patch-clamp recording was performed to investigate the effect of GSK1016790A or another TRPV4 activator 4α-PDD on cultured rat vagal bronchopulmonary sensory neurons. Immunohistochemistry was carried out to determine the TRPV4-expressing cells in lung slices obtained from TRPV4-EGFP mice. Our results showed, that right-atrial injection of GSK1016790A evoked a slow-developing, long-lasting rapid shallow breathing in anesthetized rats. Activation of TRPV4 also significantly potentiated capsaicin-evoked chemoreflex responses. The alteration in ventilation induced by GSK1016790A was abolished by cutting or perineural capsaicin treatment of both vagi, indicating the involvement of bronchopulmonary afferent neurons. The stimulating and sensitizing effects of GSK1016790A were abolished by a selective TRPV4 antagonist GSK2193874 and also by inhibiting cyclooxygenase with indomethacin. Surprising, GSK1016790A or 4α-PDD did not activate isolated bronchopulmonary sensory neurons, nor did they modulate capsaicin-induced inward currents in these neurons. Furthermore, TRPV4 expression was found in alveolar macrophages, alveolar epithelial, and vascular endothelial cells. Collectively, our results suggest that GSK1016790A regulates the respiration through an indirect activation of bronchopulmonary sensory neurons, likely via its stimulation of other TRPV4-expressing cells in the lungs and airways.

## Introduction

Transient receptor potential vanilloid receptor 4 (TRPV4) is a calcium-permeable non-selective cation channel that can be activated by diverse stimuli including moderate heat, shear stress, endogenous chemicals such as anandamide, arachidonic acid, and its epoxyeicosatrienoic acid metabolites, as well as by a growing number of exogenous chemical ligands such as 4α-PDD and GSK1016790A (Vriens et al., 2004; Heller and O'Neil, 2007; Nilius and Voets, 2013). Growing evidence has shown that TRPV4 is involved in numerous physiological functions including body osmoregulation and noxious mechanical and thermal sensation, and mutation in the TRPV4 gene is known to cause skeletal dysplasia and neurodegenerative diseases (Heller and O'Neil, 2007; Nilius and Voets, 2013; Garcia-Elias et al., 2014). In the respiratory system, however, the function of TRPV4 is not well understood other than its involvement in acute lung injury (Alvarez et al., 2006; Hamanaka et al., 2010; Balakrishna et al., 2014).

Although, previous studies showed that the commonly used TRPV4 activator 4α-PDD activates a majority of dorsal root ganglion (DRG) neurons, a recent study questioned the specificity of 4α-PDD and concluded that its effect on DRG neurons is independent of TRPV4 activation (Alexander et al., 2013). On the other hand, GSK1016790A is recently identified as a selective TRPV4 channel agonist which is ~300-fold more potent than 4α-PDD (Thorneloe et al., 2008). In the present study, we aimed to investigate the ventilatory effect of GSK1016790A in anesthetized rats and further explore the potential involvement of activation of bronchopulmonary sensory neurons. Our results showed, that activation of TRPV4 may indirectly enhance the excitability of these neurons and subsequently affect the respiration through chemoreflexes.

## Materials and methods

The procedures described below were performed in compliance with the Public Health Service Policy on Humane Care and Use of Laboratory Animals (Office of Laboratory Animal Welfare, Amended August, 2002) and U.S. Government Principles for the Utilization and Care of Vertebrate Animals Used in Testing, Research, and Training. These procedures were also approved by the Institutional Animal Care and Use Committee of Mercer University and Washington University.

### Animal preparation

Adult Sprague–Dawley rats were anesthetized with an intra-peritoneal injection of α-chloralose (100 mg/kg) and urethane (500 mg/kg) dissolved in a 2% borax solution. Supplemental doses (one tenth of the initial dose) of the same anesthetics were injected intravenously to maintain abolition of pain reflexes induced by tail pinch. The left jugular venous catheter was cannulated with its tip positioned slightly above the right atrium for bolus injection of chemical stimuli. The left femoral artery was cannulated for recording arterial blood pressure (ABP). In some animals, the right femoral vein was also cannulated for infusion of pharmacological agents. A short tracheal cannula was inserted just below the larynx via a tracheotomy. Body temperature was maintained at 37°C throughout the experiment by means of heating pad placed under the animal lying in a supine position. At the end of experiment, animals were euthanized by intravenous injection of an over dose of 3 M KCl.

### *In-vivo* cardiopulmonary recordings

Animals breathed spontaneously through the tracheal cannula. Respiratory flow was measured by a differential pressure transducer (HSE-HA DLP2.5, Harvard Apparatus, Holliston, MA). Respiratory frequency (RF), tidal volume (V_T_), expiratory duration (T_E_), ABP, and heart rate (HR) were recorded and analyzed using PowerLab and LabChart Pro from ADInstruments (Colorado Springs, CO). Pulmonary chemoreflex responses were measured when intravenous injections of capsaicin (0.75–1.25 μg/kg), a selective transient receptor potential vanilloid receptor 1 (TRPV1) agonist, were administered. At least 15 min was allowed to elapse between two consecutive injections to avoid tachyphylaxis. To determine the intensity of apneic response, the apneic ratio was calculated by dividing the longest T_E_ occurring within 3 s after the injection by the baseline T_E_ that was averaged over 10 breaths immediately preceding the injection.

### Retrograde labeling of vagal bronchopulmonary sensory neurons

Sensory neurons innervating airways and lungs were identified by retrograde labeling using the fluorescent tracer 1,1′-dioctadecyl-3,3,3′,3′-tetramethylindocarbocyanine perchlorate (DiI), as described in our recent studies (Gu et al., 2013). Briefly, young Sprague–Dawley rats (80–120 g) were anesthetized with continuous inhalation of isoflurane administered via a nose cone connected to a vaporizing machine (Smiths Medical, Dublin, OH). A small mid-line incision was made on the ventral neck skin to expose the trachea. DiI (0.2 mg/ml, 50 μl) was instilled into the lungs via a 30-gage needle inserted into the lumen of the trachea; the skin incision was then closed. Animals were kept undisturbed for 7–10 days until they were used for tissue harvest.

### Isolation of nodose and jugular ganglion neurons

Rats were decapitated after being anesthetized by isoflurane inhalation. The head was immediately immersed in ice-cold Dulbecco's modified Eagle's medium (DMEM)/F12 solution, followed by quick extraction of nodose and jugular ganglia under a dissecting microscope. Each ganglion was desheathed, cut into eight pieces, placed in a 0.08% type IV collagenase, and incubated for 60 min in 5% CO_2_ in air at 37°C. The ganglion suspension was centrifuged (150 g, 5 min) and supernatant aspirated. The cell pellet was resuspended in 0.05% trypsin for 1 min and centrifuged (150 g, 5 min); the pellet was then resuspended in a modified DMEM/F12 solution (supplemented with 10% heat-inactivated fetal bovine serum, 100 units/ml penicillin, 100 μg/ml streptomycin, and 100 μM minimum essential media nonessential amino acids) and gently triturated with a small-bore fire-polished Pasteur pipette. Myelin debris was separated and discarded after centrifugation of the dispersed cell suspension (500 g, 8 min) through a layer of 15% bovine serum albumin. The cell pellet was resuspended in the modified DMEM/F12 solution, plated onto poly-L-lysine-coated glass coverslips, and incubated overnight (5% CO_2_ in air at 37°C).

### Whole-cell perforated patch-clamp recordings

Whole-cell perforated (50 μg/ml gramicidin) patch-clamp recordings were carried out using Axopatch 200B, Digidata 1440A, and pCLAMP 10 software (Molecular Devices, Sunnyvale, CA), as described previously (Gu et al., 2013). The recording chamber with cultured cells was perfused continuously with the standard extracellular solution (ECS) containing in mM: 136 NaCl, 5.4 KCl, 1.8 CaCl_2_, 1 MgCl_2_, 0.33 NaH_2_PO_4_, 10 glucose, 10 HEPES, pH at 7.4. The intracellular solution contained (in mM): 92 potassium gluconate, 40 KCl, 8 NaCl, 1 CaCl_2_, 0.5 MgCl_2_, 10 EGTA, 10 HEPES, pH at 7.2. The chemical stimulants were applied by a pressure-driven drug delivery system (VC^3^8, ALA Scientific Instruments, Westbury, NY). The resting membrane potential was held at −70 mV. The series resistance was usually in the range of 6–10 MΩ and was not compensated. The experiments were performed at room temperature (~22°C). Data from nodose and jugular ganglion neurons were pooled for group analysis since no difference was found between responses of the neurons obtained from these two ganglia.

### Immunohistochemistry

Bacterial artificial chromosome (BAC)-transgenic Tg(TRPV4-EGFP)MT43Gsat mice (designated as TRPV4^eGFP^) were purchased from Mutant Mouse Resource Research Centers. TRPV4-expressing cells were identified by expressing the eGFP protein under control of the TRPV4 promoter (Gong et al., 2003). Adult mice of 6–16 weeks were asphyxiated with CO_2_ and perfused transcardially with 200 ml of 30% sucrose in 0.1 M phosphate buffer (PB; pH 7.3) followed by 200 ml of fixative [4% paraformaldehyde or Zamboni's fixative (2% paraformaldehyde, 15% saturated picric acid) in 0.1 M PB, pH 7.3]. The lungs were removed and post-fixed overnight at 4°C in the same fixative. All tissues were cryoprotected overnight in 30% sucrose in 0.1 M PB, pH 7.3, frozen in optimal cutting temperature medium, sectioned with a cryostat at 20 μm, mounted on Superfrost Plus slide, and stored at −20°C. Frozen slides were dried at room temperature for 1 h and washed three times in PBS with 0.1% Triton X-100 (PBS+TX), blocked for 30 min to 1 h in PBS+TX containing 10% donkey serum, and incubated overnight at 4°C with primary antibodies diluted in blocking solution: chicken anti-GFP (1:500; Aves Labs, Tigard, OR), and rat anti-F4/80 (1:500; Biolegend, San Diago, CA). The sections were then washed three times in PBS+TX and incubated for 2 h at room temperature with secondary antibodies conjugated to Alexa-488 fluorochrome (Life Technologies, Grand Island, NY) for chicken anti-GFP or Cy3 fluorochromes (Jackson ImmunoResearch, West Grove, PA) for rat anti-F4/80, and diluted 1:500 in blocking solution. Sections were then washed three times in PBS+TX and mounted with anti-fade medium Vectashield (Vector Laboratories, Burlingame, CA). All preparations were examined with Nikon A1 Confocal Laser Microscope System. Images were taken and analyzed with Nikon NIS-Elements software.

### Chemicals

All chemicals were purchased from Sigma-Aldrich (St. Louis, MO). A stock solution of capsaicin (1 mM) was prepared in 1% Tween 80, 1% ethanol, and 98% saline. Stock solutions of GSK1016790A, 4α-PDD and GSK2193874 (10 mM) were prepared in dimethyl sulfoxide, aliquoted and kept at −80°C. Chemicals at desired concentrations were prepared daily by dilution with isotonic saline in *in-vivo* studies or with ECS in patch-clamp recordings. Indomethacin and Nω-nitro-L-arginine methyl ester (L-NAME) were freshly prepared before experiments in 0.1 M NaHCO_3_ and isotonic saline, respectively. No detectable effect of vehicles of chemical agents was found in our preliminary experiments.

## Statistical analysis

One-way repeated-measure ANOVA was used, unless mentioned otherwise, for statistical analysis with SigmaPlot 12 (Systat Software, San Jose, CA). When the ANOVA showed a significant interaction, pair-wise comparisons were made with the Fisher's least significant difference *post-hoc* analysis. Data are presented as the mean ± s.e.m. A value of *p* < 0.05 was considered to be significant.

## Results

### GSK1016790A evoked rapid shallow breathing in anesthetized spontaneously breathing rats

**Figure 1:**
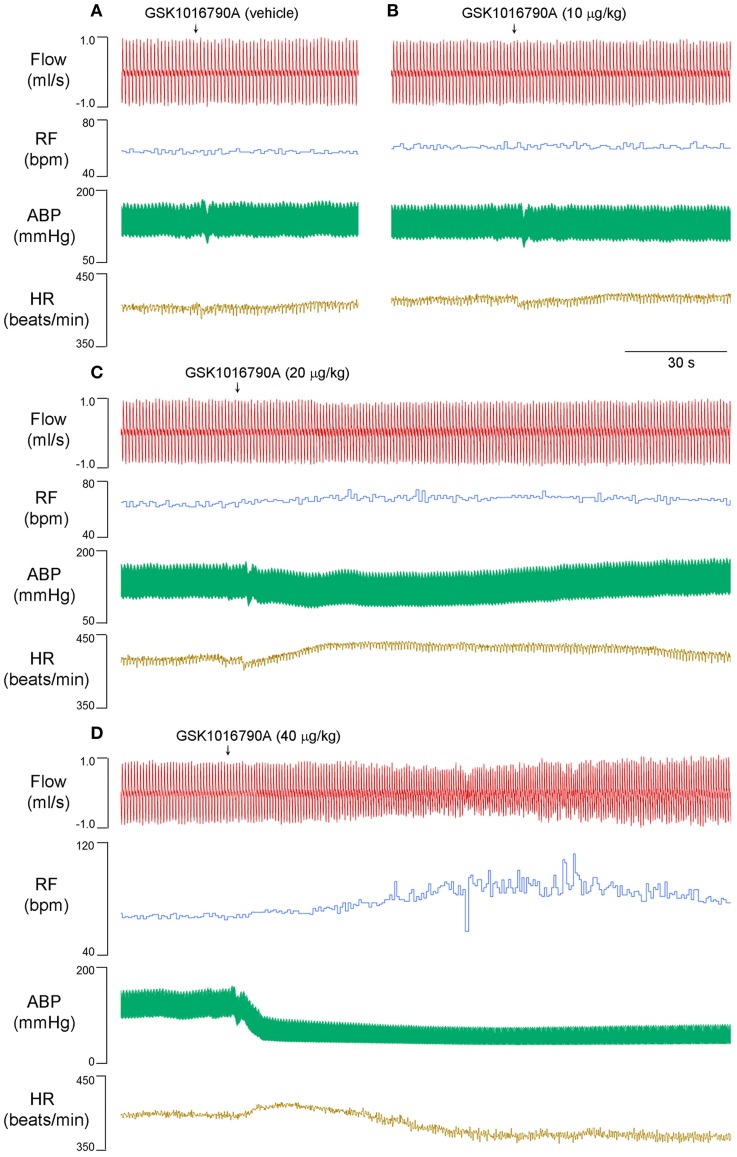
**Right atrial injection of GSK1016790A dose-dependently evoked rapid shallow breathing in anesthetized spontaneously breathing rats**. Injectate of GSK1016790A (0–40 μg/kg in 0.15 ml) was first slowly injected into the catheter (dead space volume 0.2 ml) and then flushed (at arrow), as a bolus with saline (0.3 ml). Note that **(A–C)** were recorded from one animal, and **(D)** from another animal to show a clean recording. Flow, respiratory flow; RF, respiratory frequency; ABP, arterial blood pressure; HR, heart rate; bpm, breath per minute.

Right atrial injection of GSK1016790A did not induce a typical apnea, but it evoked rapid shallow breathing that developed rather slowly, peaked within 2 min, and lasted up to 10 min after the injection (Figure 1). For example, 40 μg/kg GSK1016790A significantly increased the respiratory frequency from 62.7 ± 1.9 to 79.6 ± 3.2 breath per min and reduced tidal volume from 1.9 ± 0.0 to 1.5 ± 0.1 ml (*n* = 5; *p* < 0.01), whereas the minute ventilation was not significantly altered after GSK1016790A (*n* = 5; *p* > 0.05; Table 1). At both 20 and 40 μg/kg, GSK1016790A also evoked a significant decrease in arterial blood pressure, and increase in heart rate (Figure 1; Table 1).

**Table 1:** **Cardiopulmonary effect of GSK1016790A in anesthetized rats**.

**Parameters**	**Baseline (*n* = 20)**	**GSK1016790A (vehicle; *n* = 5)**	**GSK1016790A (10 μg/kg; *n* = 5)**	**GSK1016790A (20 μg/kg; *n* = 5)**	**GSK1016790A (40 μg/kg; *n* = 5)**
RF (bpm)	62.7±1.9	62.1±5.2	63.3±3.5	66.7±3.8	79.6±3.2^**^
V_T_ (ml)	1.9±0.0	1.9±0.1	1.9±0.1	1.9±0.1	1.5±0.1^**^
MV (ml/min)	116.2±2.6	115.2±7.8	117.5±3.1	126.1±3.0	115.8±4.4
ABP (mmHg)	126.7±3.2	124.9±6.8	127.9±4.2	97.2±10.0^**^	57.4±2.5^**^
HR (beats/min)	395.3±9.1	382.5±23.3	419.8±6.4	433.8±4.4^*^	431.4±11.5^*^

*The baseline parameters were calculated as the average values over 20-s duration immediately before the injection of GSK1016790A; the parameters after GSK1016790A were calculated as the average values over 20-s duration after the administration of GSK1016790A when changes in these parameters were the greatest from corresponding baseline values. RF, respiratory frequency; V_T_, tidal volume; MV, minute ventilation; ABP, arterial blood pressure; HR, heart rate; bpm, breath per minute*.

* p < 0.05 and

** *p < 0.01, significantly different from corresponding baseline values; Student's t-test*.

### GSK1016790A potentiated capsaicin-evoked pulmonary chemo-reflexes in anesthetized rats

**Figure 2:**
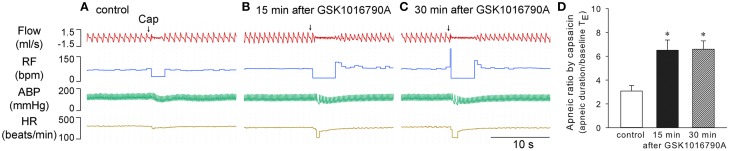
**Potentiation of capsaicin-evoked chemoreflexes by GSK1016790A in anesthetized rats**. **(A–C)** Responses to right atrial injection of capsaicin (1.0 μg/kg) before, and 15 and 30 min after administration of GSK1016790A (40 μg/kg, i.v.). **(D)** Effect of GSK1016790A on the reflex apneic response to capsaicin (0.75–1 μg/kg). Apneic duration, longest expiratory duration within 3 s after injection of capsaicin; baseline *T*_E_, average expiratory duration over 10 consecutive breaths immediately preceding the capsaicin injection. ^*^Significantly different from the control response (*n* = 5; *p* < 0.05).

Capsaicin is known to evoke apnea, bradycardia, and hypotension through a stimulation of TRPV1 channels in bronchopulmonary sensory neurons (Gu et al., 2005). At 15 and 30 min after 40 μg/kg GSK1016790A administration, when the cardiopulmonary changes induced by GSK1016790A already returned to baseline values, the capsaicin-evoked chemoreflexes were significantly potentiated. The apneic ratio evoked by capsaicin was increased from 3.1 ± 0.5 at control to 6.5 ± 0.9 and 6.6 ± 0.7 at 15 and 30 min after GSK1016790A, respectively, (*n* = 5; *p* < 0.05; Figure 2).

### TRPV4 inhibition prevented cardiopulmonary effects of GSK1016790A

**Figure 3:**
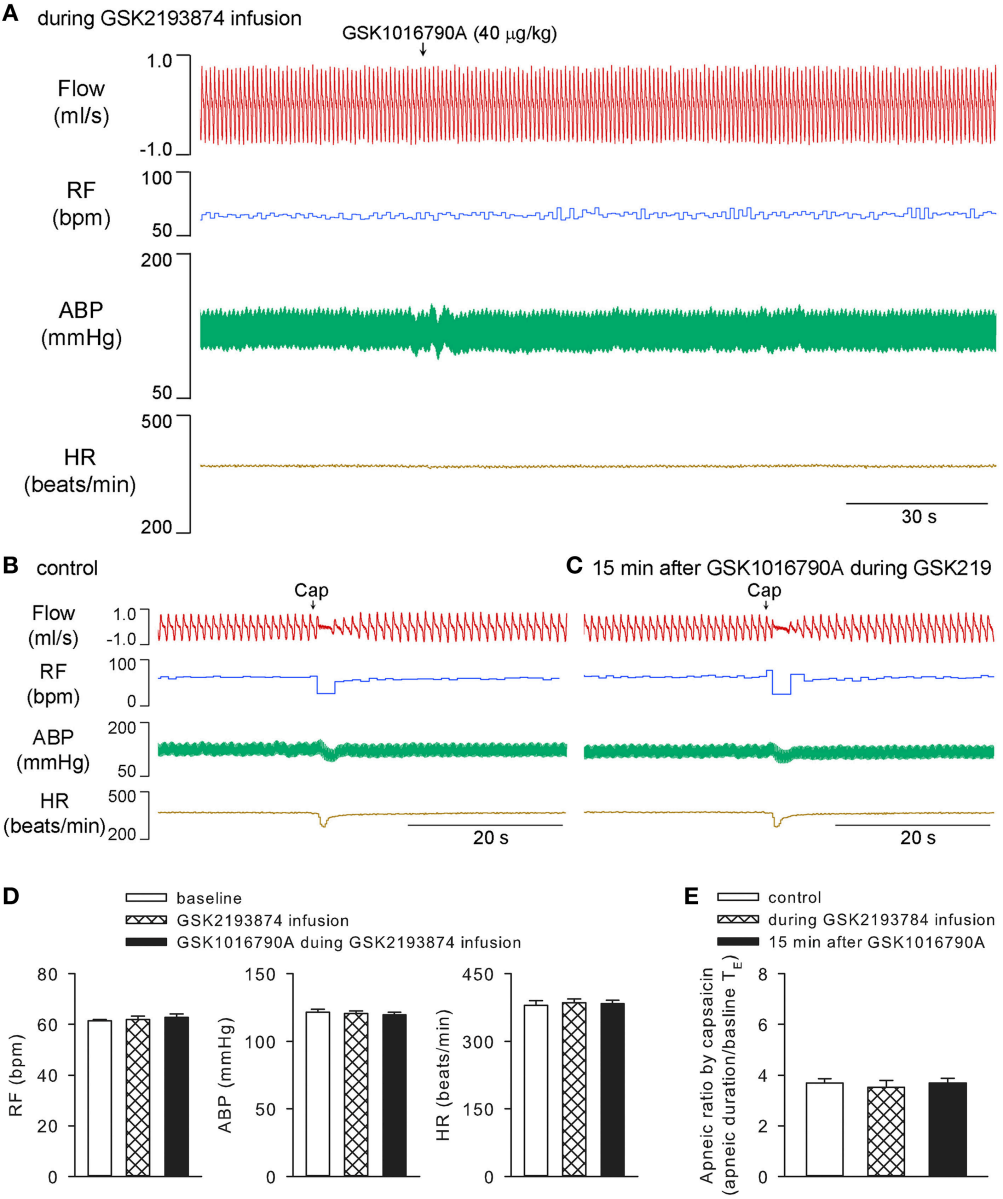
**Cardiopulmonary effects of GSK1016790A were abolished by specific TRPV4 antagonist GSK2193874 in anesthetized rats**. GSK2193874 (0.2 mg/kg) was systemically infused for 30 min at a constant rate of 0.1 ml/min. GSK1016790A (40 μg/kg) was administered at 15 min after start of GSK2193874 infusion **(A)**. Capsaicin-evoked chemoreflexes were recorded at control **(B)**, and 15 min after GSK1016790A administration **(C)**. Group data show that GSK1016790A did not induce significant change in RF, ABP, or HR **(D)**, nor did its pretreatment alter the capsaicin-evoked apneic response **(E)**, during GSK2193874 infusion (*n* = 5; *p* > 0.05). Note, that to avoid tachyphylaxis, chemoreflexes evoked by capsaicin during GSK2193874 infusion, the middle column shown in **(E)**, were carried out in separate group of animals.

Systemic infusion of potent and selective TRPV4 antagonist GSK2193874 (Thorneloe et al., 2012; 0.2 mg/kg, 0.1 ml/min for 30 min) had no significant effect on baseline cardiopulmonary values; however, it completely prevented the GSK1016790A-induced changes in breathing, blood pressure, or heart rate, as well as its potentiation of capsaicin-evoked chemoreflex responses (*n* = 5; *p* > 0.05; Figure 3).

### Involvement of vagal sensory nerves in the ventilatory effect of GSK1016790A

**Figure 4:**
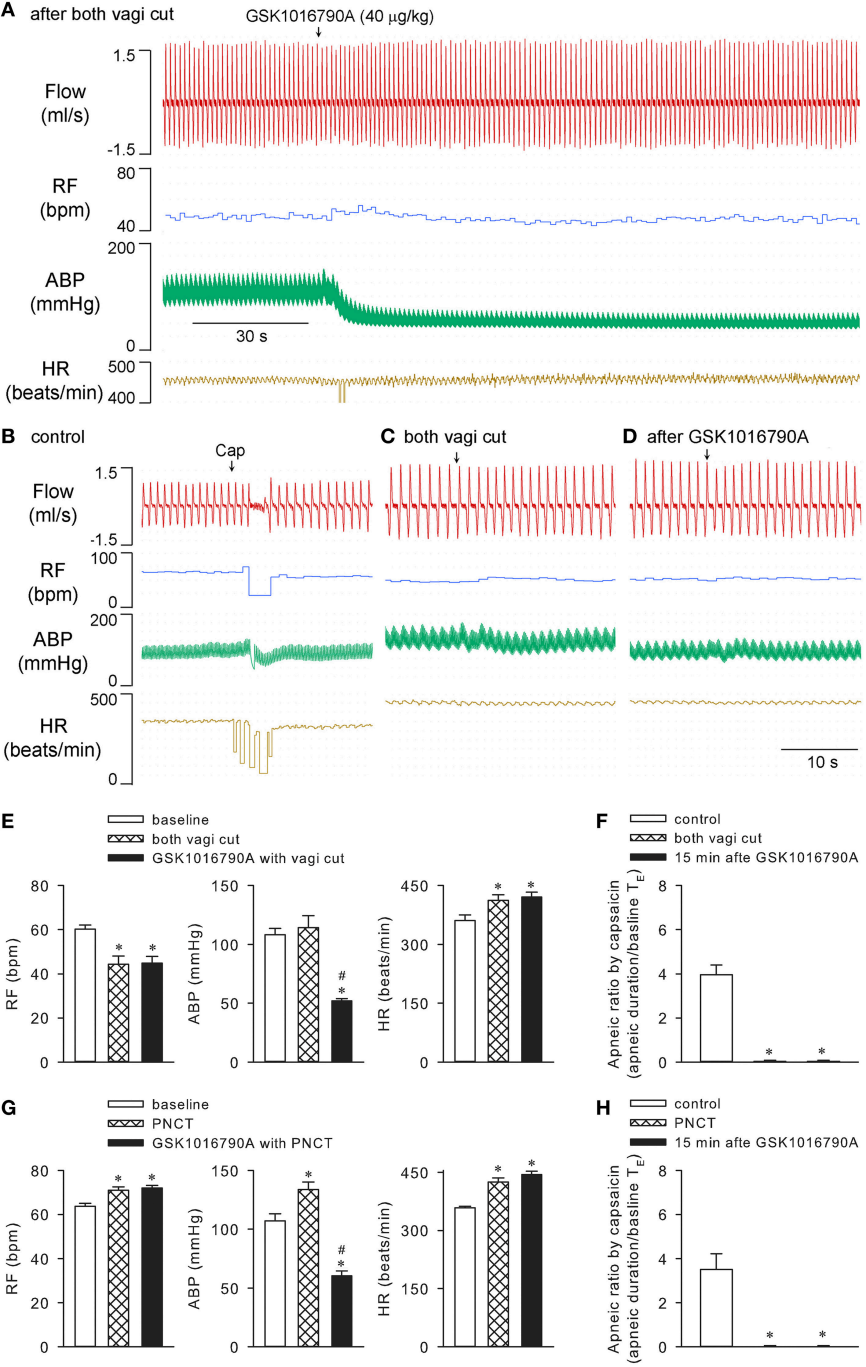
**Cutting or perineural capsaicin treatment of both vagi effectively abolished effects of GSK1016790A on respiration in anesthetized rats**. **(A)** GSK1016790A (40 μg/kg) injection after both vagi cut. **(B–D)** Capsaicin-evoked responses at control, before and 15 min after GSK1016790A administration with vagi cut. **(E,F)** Group data show GSK1016790A-induced changes in RF, ABP, HR, and capsaicin-evoked apneic response, respectively, before and after vagi cut. **(G,H)** Group data show GSK1016790A-induced changes in RF, ABP, HR, and capsaicin-evoked apneic response, respectively, before and after bilateral perineural capsaicin treatment (PNCT; 300 μg/ml capsaicin). The latter was applied to both vagi 20 min before GSK1016790A administration. ^*^Significantly different from the corresponding baseline control; #significantly different from corresponding values after vagi cut (*n* = 5; *p* < 0.05) or PNCT (*n* = 4; *p* < 0.05) alone.

Cutting both vagi prevented the rapid shallow breathing (*n* = 5; *p* > 0.05), but not the hypotension (*n* = 5; *p* < 0.05) evoked by 40 μg/kg GSK1016790A. Not surprisingly, cutting vagi completely abolished the capsaicin-evoked chemoreflexes both before and after GSK1016790A (*n* = 5; *p* > 0.05; Figures 4A–F). Similarly, both stimulating and sensitizing effects of GSK1016790A on ventilation were also effectively prevented with perineural capsaicin treatment of both vagi which presumably blocks the conductance of bronchopulmonary C-fibers (*n* = 4; Figures 4G,H), indicating the involvement of activation of bronchopulmonary sensory afferents (Gu et al., 2005).

### Ventilatory effect of GSK1016790A was abolished by systemic infusion of indomethacin but not L-NAME

**Figure 5:**
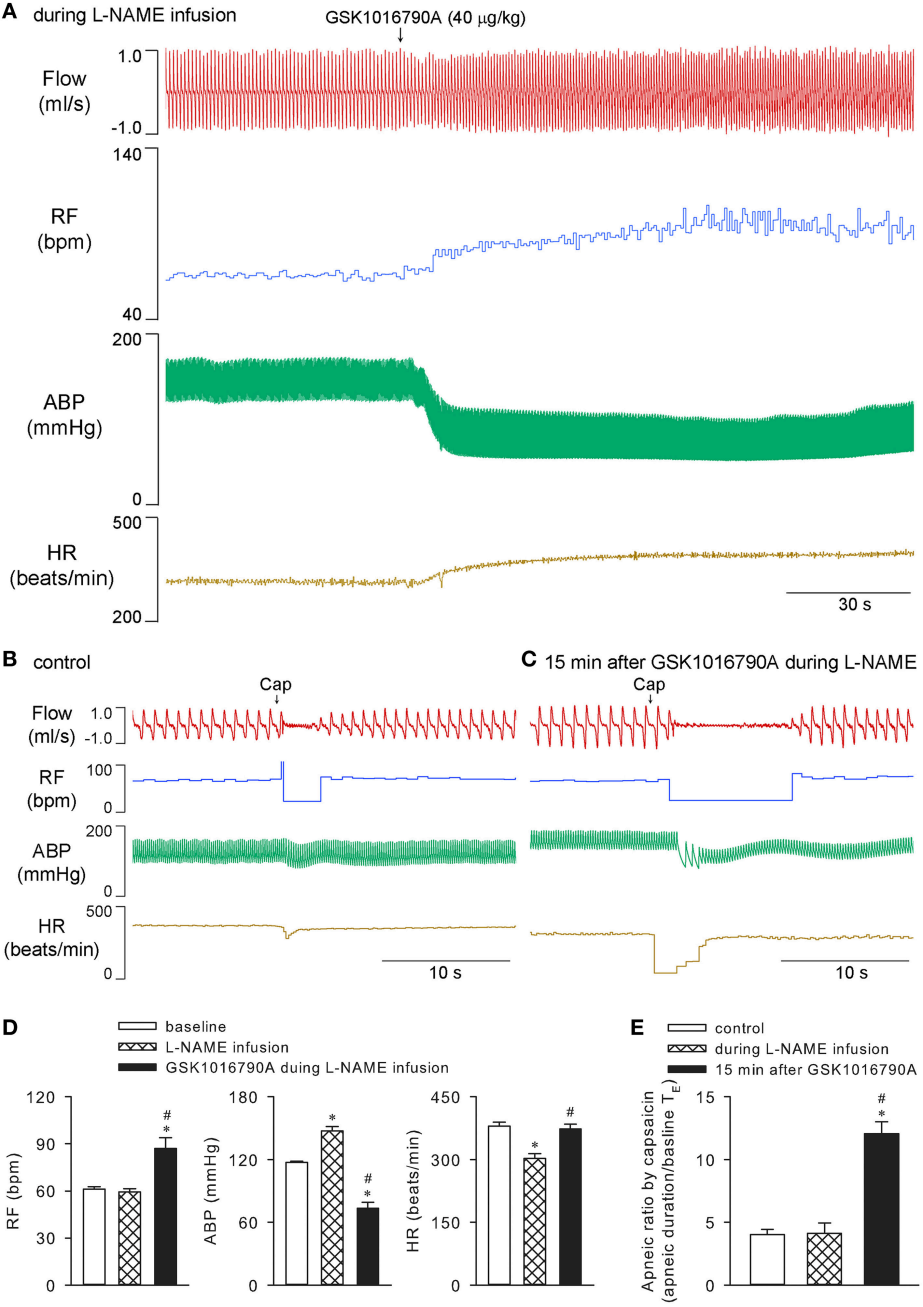
**Cardiopulmonary effects of GSK1016790A during systemic infusion of L-NAME in anesthetized rats**. Nitric oxide synthesis inhibitor L-NAME (10 mg/kg) was constantly infused via a femoral vein at a rate of 0.1 ml/min for 30 min. GSK1016790A (40 μg/kg) was administered at 15 min after start of L-NAME infusion **(A)**. Capsaicin-evoked chemoreflexes were recorded at control **(B)**, and 15 min after GSK1016790A administration and right before the termination of L-NAME infusion **(C)**. Group data show GSK1016790A-induced changes in RF, ABP, and HR **(D)**, and potentiation of capsaicin-evoked apneic response **(E)**, during L-NAME infusion. ^*^Significantly different from the baseline control; ^#^significantly different from values during L-NAME infusion (*n* = 5; *p* < 0.05).

It was reported previously, that the GSK1016790A-induced relaxation of isolated vessels is probably endothelial/nitric oxide dependent (Köhler et al., 2006; Willette et al., 2008). Systemic constant infusion of 10 mg/kg L-NAME (0.1 ml/min for 30 min), a dose known to effectively inhibit nitric oxide synthesis in rats (Badejo et al., 2008), was administered to test whether it affected the observed cardiopulmonary effects of GSK1016790A in our preparation. As expected, L-NAME infusion alone significantly elevated arterial blood pressure and reduced heart rate (*n* = 5; *p* < 0.05; e.g., Figure 5D), while not affecting capsaicin-evoked apneic responses (*n* = 5; *p* > 0.05; e.g., Figure 5E). It did not inhibit 40 μg/kg GSK1016790A-induced alterations in breathing, blood pressure, or heart rate (*n* = 5; *p* < 0.05). L-NAME also failed to abolish the potentiation of capsaicin-evoked chemoreflexes by GSK1016790A (*n* = 5; *p* < 0.05; Figure 5).

**Figure 6:**
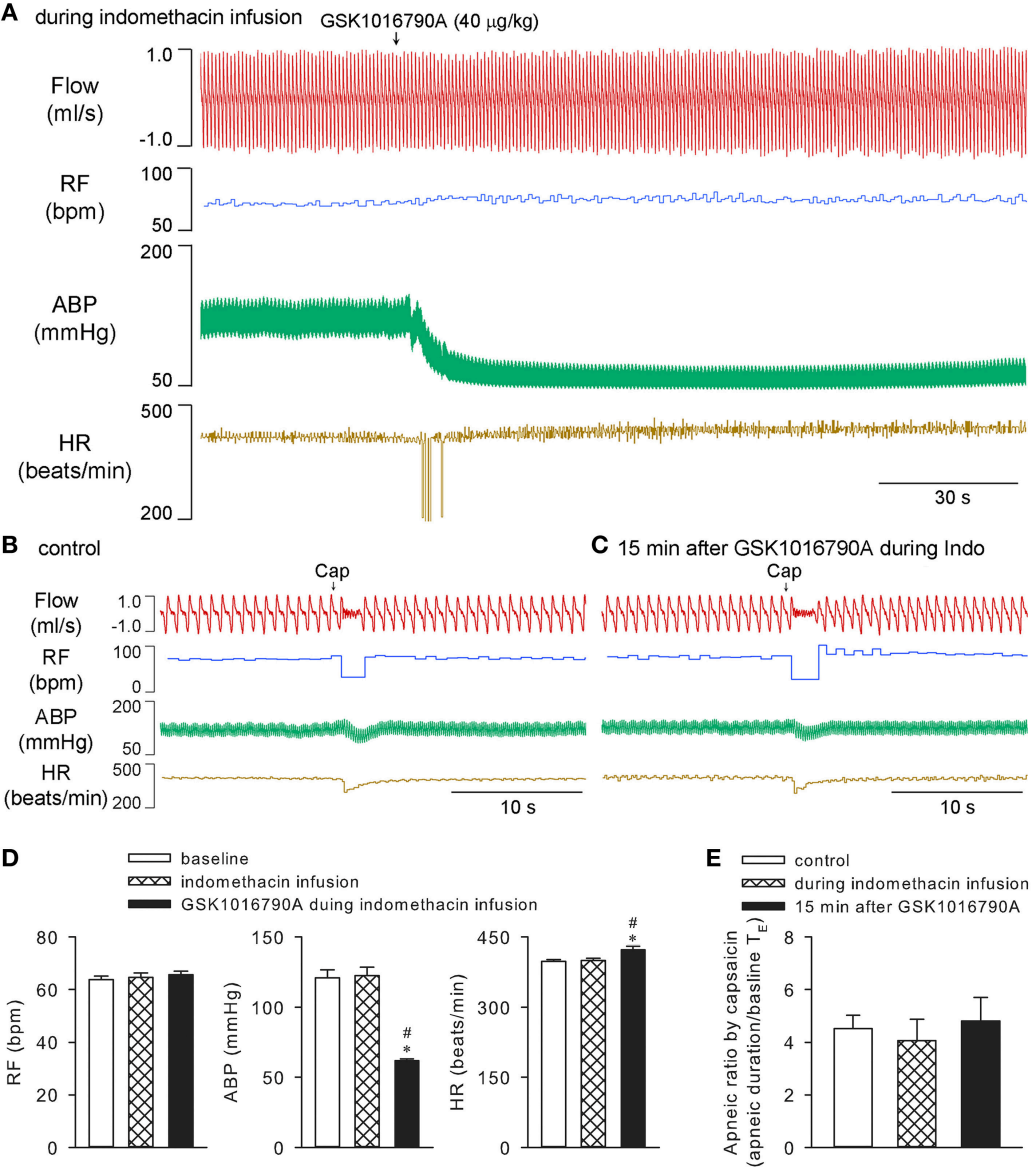
**Effects of GSK1016790A on ventilation were inhibited by indomethacin in anesthetized rats**. Cyclooxygenase inhibitor indomethacin (10 mg/kg) was constantly infused at a rate of 0.1 ml/min for 30 min. GSK1016790A (40 μg/kg) was administered at 15 min after start of indomethacin infusion **(A)**. Capsaicin-evoked chemoreflexes were recorded at control **(B)**, and 15 min after GSK1016790A administration **(C)**. Group data show that GSK1016790A induced hypotension and increase in heart rate **(D)**, but not significant change in respiratory rate **(D)** or capsaicin-evoked apneic response **(E)**, during indomethacin infusion. ^*^Significantly different from the baseline control; ^#^significantly different from values during indomethacin infusion (*n* = 5; *p* < 0.05).

On the other hand, infusion of indomethacin in the same manner (10 mg/kg, 0.1 ml/min for 30 min) which was known to effectively inhibit the cyclooxygenase in anesthetized rat preparation (Badzyńska et al., 2003), effectively prevented both stimulating and sensitizing effects of GSK1016790A on ventilation (*n* = 5; *p* > 0.05), while not affecting the hypotension induced by GSK1016790A (*n* = 5; *p* < 0.05). Indomethacin alone did not significantly affect the cardiopulmonary parameters, nor did it significantly alter capsaicin-evoked apneic responses in these animals (*n* = 5; *p* > 0.05; Figure 6).

### TRPV4 is not functionally expressed in bronchopulmonary sensory neurons

**Figure 7:**
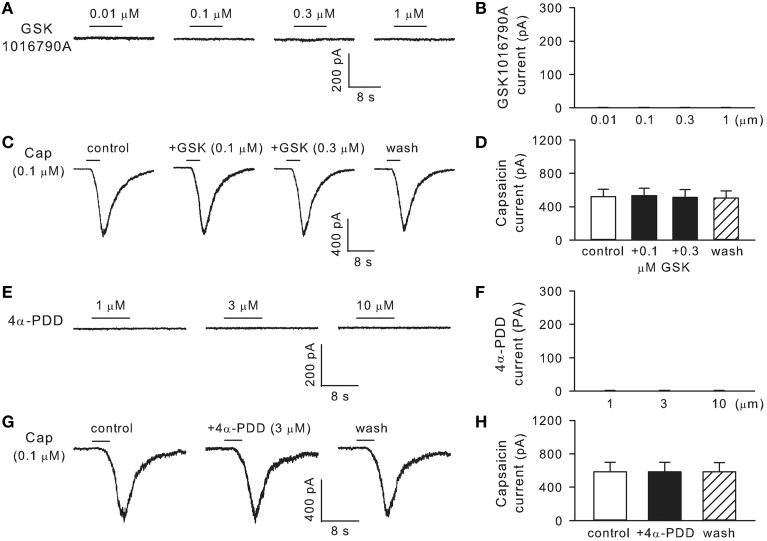
**TRPV4 is not functionally expressed in rat bronchopulmonary sensory neurons**. **(A,B)** Show that GSK1016790A (0.01–1 μM, 8 s) did not induce any whole-cell inward current (*n* = 24). **(C,D)** Show, that pretreatment with GSK1016790A (0.1 or 0.3 μM, 60 s) did not significantly affect the capsaicin-evoked TRPV1 current (*n* = 10–16; *p* > 0.05). (**E–H**) Show that 4α-PDD (1–10 μM, 8 s) did not induce any inward current (*n* = 16), nor did its pretreatment (3 μM, 60 s) significantly alter the capsaicin-evoked TRPV1 current (*n* = 11; *p* > 0.05).

Whole-cell perforated patch-clamp was carried out in isolated rat bronchopulmonary sensory neurons. In 24 neurons obtained from four different cultures including 16 capsaicin-sensitive and 8 capsaicin-insensitive neurons, GSK1016790A (0.01–1 μM, 8 s) failed to induce any whole-cell inward current (*n* = 24; Figures 7A,B). In addition, pretreatment with GSK1016790A (0.1 or 0.3 μM, 60 s) did not significantly affect the capsaicin (0.1 or 0.3 μM, 3–10 s) -evoked TRPV1 current (*n* = 10–16; *p* > 0.05; Figures 7C,D). Similarly, in a separate group of 16 neurons including 11 capsaicin-sensitive and 5 capsaicin-insensitive ones, the commonly used TRPV4 activator 4α-PDD (1–10 μM, 8 s) failed to induce any whole-cell current (*n* = 16), nor did its pretreatment (3 μM, 60 s) significantly alter the capsaicin (0.1 or 0.3 μM, 3–10 s) -evoked inward current in these neurons (*n* = 11; *p* > 0.05; Figures 7E–H).

### Expression of TRPV4 in the lung

**Figure 8:**
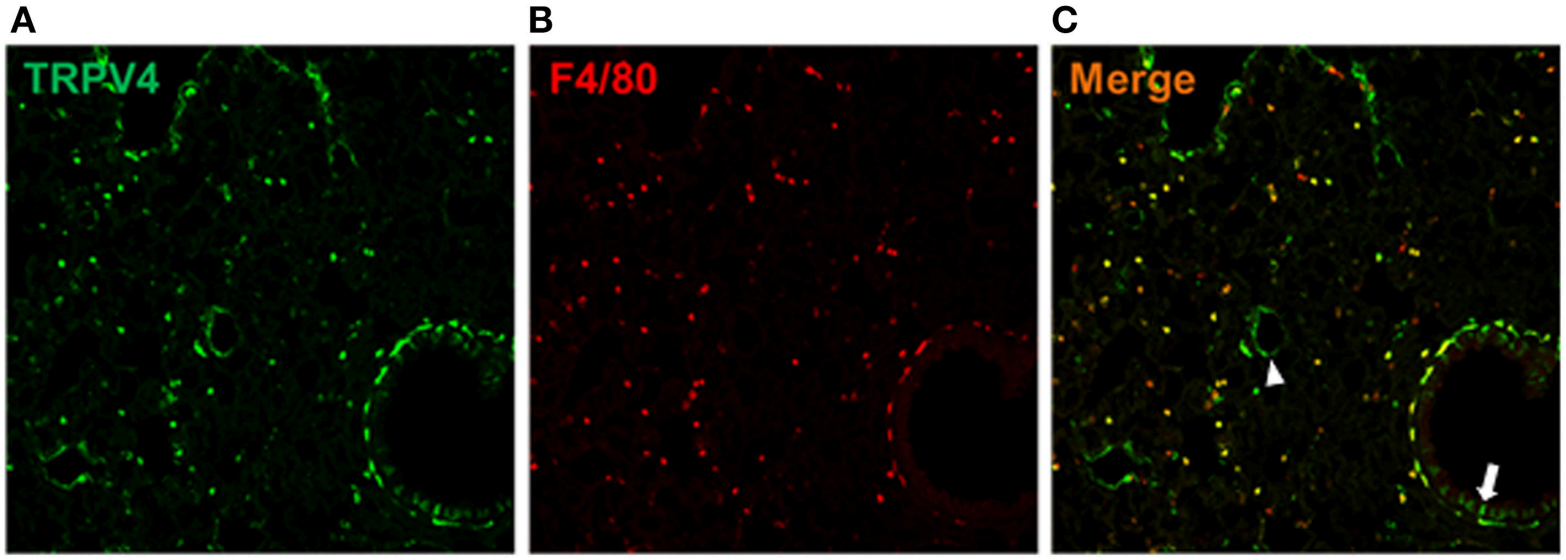
**TRPV4-expressing cells in the lung**. The TRPV4^eGFP^ cells **(A)** expressed a pan macrophage marker F4/80 **(B)**. The merged image **(C)** showed, that TRPV4^eGFP^ was also expressed by vascular endothelial cells (arrow head) and alveolar epithelial cells (arrow) in the mouse lung.

We examined the TRPV4^eGFP^-expressing cells in the TRPV4 BAC-transgenic mice. Interestingly, the TRPV4^eGFP^ is shown in alveolar macrophages expressing a pan macrophage marker F4/80. In addition, TRPV4 expression is also found in vascular endothelial cells and alveolar epithelial cells shown in mouse lung sections (Figure 8).

## Discussion

Our results showed that right atrial injection of GSK1016790A induced rapid shallow breathing which developed slowly and lasted up to 10 min. GSK1016790A also had long-lasting potentiating effect on capsaicin-evoked chemoreflexes. The ventilatory effects of GSK1016790A were effectively prevented by cutting or perineural capsaicin treatment of both vagi, indicating involvement of bronchopulmonary sensory nerves. However, our study showed that TRPV4 was not functionally expressed in these sensory neurons, which is surprising considering TRPV4 mRNA and proteins were found in these neurons previously by means of RT-PCR and immunohistochemistry (Ni et al., 2006). Similarly, although expression and activation of TRPV4 in the peripheral nervous system has long been implicated in thermal and mechanical hyperalgesia as well as in pressure and heat sensation (Todaka et al., 2004; Alessandri-Haber et al., 2006, 2008; Vergnolle et al., 2010; Chen et al., 2013), a recent study by Alexander et al. (2013) questioned the existence of functional TRPV4 channels in the vast majority of cultured DRG and trigeminal ganglion neurons. The authors offered several possible explanations for the discrepancy: (1) usage of non-selective antibodies for TRPV4; (2) inappropriate usage of 4α-PDD as a selective TRPV4 activator; and (3) misinterpretation of TRPV4 activity in non-neuronal cells as its neuronal function. Whether these possibilities also attribute to the discrepancy between our study and the previous report (Ni et al., 2006) is not yet clear.

Results of our patch-clamp study showed, that GSK1012790A or 4α-PDD did not induce any inward current in isolated capsaicin-sensitive or capsaicin-insensitive bronchopulmonary sensory neurons, nor did these two TRPV4 activators affect the TRPV1 current in capsaicin-sensitive neurons, suggesting that TRPV4 is not functionally expressed in bronchopulmonary afferents including unmyelinated C-fibers and myelinated stretch receptors. The *in-vivo* cardiopulmonary effects of GSK1016790A were mediated through activation of TRPV4 as evidenced by a complete blockade by specific TRPV4 antagonist GSK2193874. Interestingly, both stimulatory and sensitizing effects of GSK1016790A on ventilation were also effectively prevented by systemic infusion of a non-selective cyclooxygenase inhibitor indomethacin, indicating an involvement of some inflammatory mediators downstream of cyclooxygenase pathway including prostaglandins, prostacyclin and thromboxanes. Such mediators such as prostaglandin E_2_ have been previously shown to play an important role in regulating the excitability of bronchopulmonary sensory terminals and inducing cough (Ho et al., 2000; Gu et al., 2003; Maher et al., 2009). In addition, all TRPV4-expressing cells in the lungs shown in our study, including macrophages, epithelial, and endothelial cells, are known to be cell sources for release of prostaglandins upon stimulation (Liu et al., 2007; Profita et al., 2010; Giles et al., 2012).

The effects of TRPV4 activation on breathing shown in our study seems consistent with the effects of stimuli that evoke bronchoconstriction, which in turn would activate rapidly adapting stretch recpeotrs (Chou et al., 2008). A recent study using isometric tension measurements in *ex-vivo* airways showed, that activation of TRPV4 by GSK1016790A caused human airway constriction (McAlexander et al., 2014). The authors further suggested that the constriction was dependent upon the production of cysteinyl leukotrienes, probably from mast cells situated near the bronchial smooth muscle. This latter finding could reflect a species difference though since leukotrienes such as leukotriene C4 and leukotriene D4 are known not to regulate rat airway smooth muscle (Henry et al., 1992). An alternate explanation for the changes in respiration induced by TRPV4 activation is that it could simply being driven by the pulmonary edema elicited by GSK1016790A. However, the study by Willette et al. (2008) seems argue against this hypothesis because although GSK1016790A at a high dose of 0.3 mg/kg/15 min caused drastic pulmonary edema and congestion associated with perivascular and alveolar hemorrhage, such abnormalities were not detected when a dose of GSK1016790A (30–100 μg/kg, i.v.) comparable to what we used in this study was administered.

TRPV4 has been identified recently as an important player in ventilator- and chemical-induced acute lung injury. It is suggested, that TRPV4-mediated Ca^2+^-influx into alveolar epithelial and vascular endothelial cells contributes to barrier disruption and pulmonary edema (Alvarez et al., 2006). The hypothesis was further supported by findings that TRPV4 inhibitor suppressed acid-induced pulmonary inflammation by diminishing neutrophils, macrophages, and associated chemokines and cytokines, while improving tissue pathology; these effects were recapitulated in TRPV4-deficient mice (Balakrishna et al., 2014). Studies on ventilator-induced lung injury suggested that TRPV4 channels in alveolar macrophages are crucial for induction of injury since activation of TRPV4 with 4α-PDD significantly increased intracellular calcium, superoxide, and nitric oxide production in TRPV4^+∕+^ but not TRPV4^−∕−^ macrophages, while TRPV4^+∕+^ macrophages restored susceptibility of TRPV4^−∕−^ lungs to mechanical injury (Hamanaka et al., 2010). Macrophages are the main resident inflammatory cells in airways and lungs (Balhara and Gounni, 2012). In allergic asthma, drastic airway infiltration of eosinophils as well as macrophages and other inflammatory cells has been well documented (Gough et al., 2003; Ulrich et al., 2008). It is therefore tempting to speculate that activation of TRPV4 channels in alveolar macrophages might lead to hypersensitivity of bronchopulmonary sensory terminals. Thus, a given level of stimulus may evoke a greater afferent discharge and consequently a more severe airway constriction via both central cholinergic pathway and local axon reflex (Lee and Pisarri, 2001). Indeed, symptoms known to involve activation of these sensory endings, such as cough, airway irritation, hypersecretion of mucus, and reflex bronchoconstriction, are commonly reported in asthmatic patients. Whether this TRPV4-mediated neural and immune interaction indeed plays a role in the pathogenesis of asthma remains to be investigated.

Our study showed, that intravenous injection of GSK1016790A does-dependently induced hypotension in anesthetized rats which was completely abolished by GSK2193874. The vascular effects of TRPV4 activation have been investigated previously. In isolated rat carotid artery (Köhler et al., 2006) or in isolated mouse or rat aorta (Willette et al., 2008), activation of TRPV4 induces endothelia/nitric oxide-dependent relaxation. However, neither this endothelial nitric oxide generation nor the cyclooxygenase prostaglandin seems to play a major role in TRPV4-mediated hypotensive effect in our study since the latter was not significantly affected by systemic infusion of nitric oxide synthesis inhibitor L-NAME or cyclooxygenase inhibitor indomethacin. Using 4α-PDD as the TRPV4 activator, a recent study suggested that the depressor effect of TRPV4 activation attributes to, at least in part, activation of large conductance calcium-activated potassium channels in resistant vessels and calcitonin gene related peptide receptors upon the release of this peptide from sensory nerves (Gao and Wang, 2010).

In summary, our results suggest that GSK1016790A regulates the respiration through an indirect activation of bronchopulmonary sensory neurons, likely via its stimulation of other TRPV4-expressing cells in the lungs and airways.

## Author contributions

Research design: QG and HH. Conducting experiments: QG, CM, KK, CG, and HH. Data analysis: QG and HH. Manuscript writing: QG and HH.

### Conflict of interest statement

The authors declare that the research was conducted in the absence of any commercial or financial relationships that could be construed as a potential conflict of interest.
